# Sacubitril-valsartan for the treatment of hypertension in China: A cost-utility analysis based on meta-analysis of randomized controlled trials

**DOI:** 10.3389/fpubh.2022.959139

**Published:** 2022-08-17

**Authors:** Yake Lou, Ying Yu, Jinxing Liu, Jing Huang

**Affiliations:** ^1^Department of Cardiology, The Second Affiliated Hospital of Chongqing Medical University, Chongqing, China; ^2^Department of Neurology, Beijing Tiantan Hospital, Capital Medical University, Beijing, China; ^3^Department of Interventional Neuroradiology, Beijing Tiantan Hospital, Capital Medical University, Beijing, China; ^4^Department of Cardiology, National Center for Cardiovascular Diseases, Fuwai Hospital, Chinese Academy of Medical Sciences and Peking Union Medical College, Beijing, China

**Keywords:** sacubitril-valsartan, angiotensin-neprilysin inhibitors, hypertension, meta-analysis, cost-utility analysis

## Abstract

**Background:**

Sacubitril-valsartan was recommended for heart failure (HF) and proven cost-effective in HF. Recently, sacubitril-valsartan has been recommended to treat hypertension by the Chinese expert consensus. The cost utility of sacubitril-valsartan for hypertension remains uninvestigated.

**Methods:**

A meta-analysis of randomized controlled trials (RCTs) was performed to investigate the real efficacy of sacubitril-valsartan on blood pressure, compared with angiotensin receptor blockers or placebo. A lifetime Markov model was developed to compare the cost utility of sacubitril-valsartan vs. valsartan. The primary outcome was the incremental cost-utility ratio (ICUR), representing the ratio of incremental costs to the incremental utility. The willingness-to-pay (WTP) threshold was three times of per capita gross domestic product (GDP) in China in 2021. Sacubitril-valsartan was considered cost-effective if the ICUR obtained was lower than the WTP threshold, otherwise, sacubitril-valsartanis was not cost-effective.

**Results:**

A total of 10 RCTs of 5,781 patients were included in the meta-analysis. For comparison of sacubitril-valsartan 400 mg/day vs. valsartan 320 mg/day, a reduction in blood pressure (BP) of −5.97 (−6.38, −5.56) (*p* < 0.01) was observed. Cost-utility analysis showed that for a 60-year-old patient with hypertension, if sacubitril-valsartan was prescribed as the antihypertensive agent, he had a life expectancy of 11.91 quality-adjusted life-years (QALYs) with costs of 65,066 CNY, and if valsartan was prescribed as the antihypertensive agent, the life expectancy would be 11.82 QALY with costs of 54,769 CNY; thus, an ICUR of 108,622 CNY/QALY was obtained, lower than the WTP threshold.

**Conclusion:**

Compared with valsartan, sacubitril-valsartan is more effective in reducing blood pressure and may result in more quality-adjusted life-year, although with higher costs. Sacubitril-valsartan is cost-effective for hypertension in the current China setting under the willingness-to-pay threshold of 3 times of per capita GDP.

## Introduction

Hypertension is one of the major causes of heart failure, coronary heart disease, stroke, and chronic kidney disease; blood pressure (BP) lowering can significantly reduce the incidence of the above complications (1, 2). It is estimated that there are over 1.39 billion patients suffering from hypertension worldwide (3). Even though many programs have been launched to reduce the prevalence of hypertension, the number of patients with hypertension is still rising in China (4, 5).

Sacubitril-valsartan, as a kind of angiotensin-neprilysin inhibitors, has been proven effective in heart failure (HF) and recommended as the first-line treatment for HF in the 2021 European Society of Cardiology (ESC) heart failure guidelines (6, 7). Recently, randomized controlled trials (RCT) demonstrated that sacubitril-valsartan could reduce blood pressure in patients with salt-sensitive hypertension or systolic hypertension, regardless of gender or baseline blood pressure (8–10). Meta-analysis suggested that sacubitril-valsartan could decrease about 5.43 mm Hg in mean sitting systolic blood pressure (msSBP) compared with angiotensin receptor blockers (ARBs) (11). Therefore, sacubitril-valsartan was recommended to be used in Chinese patients with hypertension in the latest expert recommendations (12).

However, whether sacubitril-valsartan should be prescribed as the common antihypertensive agent remains unclear as sacubitril-valsartan is more expensive than other antihypertensive agents. What is more, the effect of sacubitril-valsartan on antihypertension is inconsistent across different RCTs (13, 14). It is necessary for us to investigate the cost-effectiveness of sacubitril-valsartan for the treatment of hypertension based on meta-analysis of RCTs, to provide more clinical evidence on the use of sacubitril-valsartan for Chinese patients with hypertension.

## Methods

### Meta-analysis

#### Search strategy

The PubMed, Embase, and Cochrane databases were searched from inception until 10 May, to identify potential citations using keywords of “neprilysin,” “sacubitril,” “valsartan,” “LCZ696,” “angiotensin receptor-neprilysin inhibition,” “blood pressure,” and “hypertension.”

Filter for search strategy RCT was derived from the Harvard Countway Library with a sensitivity of over 99%. The search details are listed in the Supplementary materials.

#### Inclusion criteria

1. Follow-up period ≥4 weeks.
2. No history of heart failure, stroke, or coronary heart diseases.
3. Interested endpoint of blood pressure.
4. Randomized controlled trials.
5. Age is no <18-year-old.

#### Exclusion criteria

1. Animal experiments.
2. The agent in the control group is not angiotensin receptor blockers or placebo.
3. Sacubitril-valsartan in the control group.

#### Data extraction

**Figure 1:**
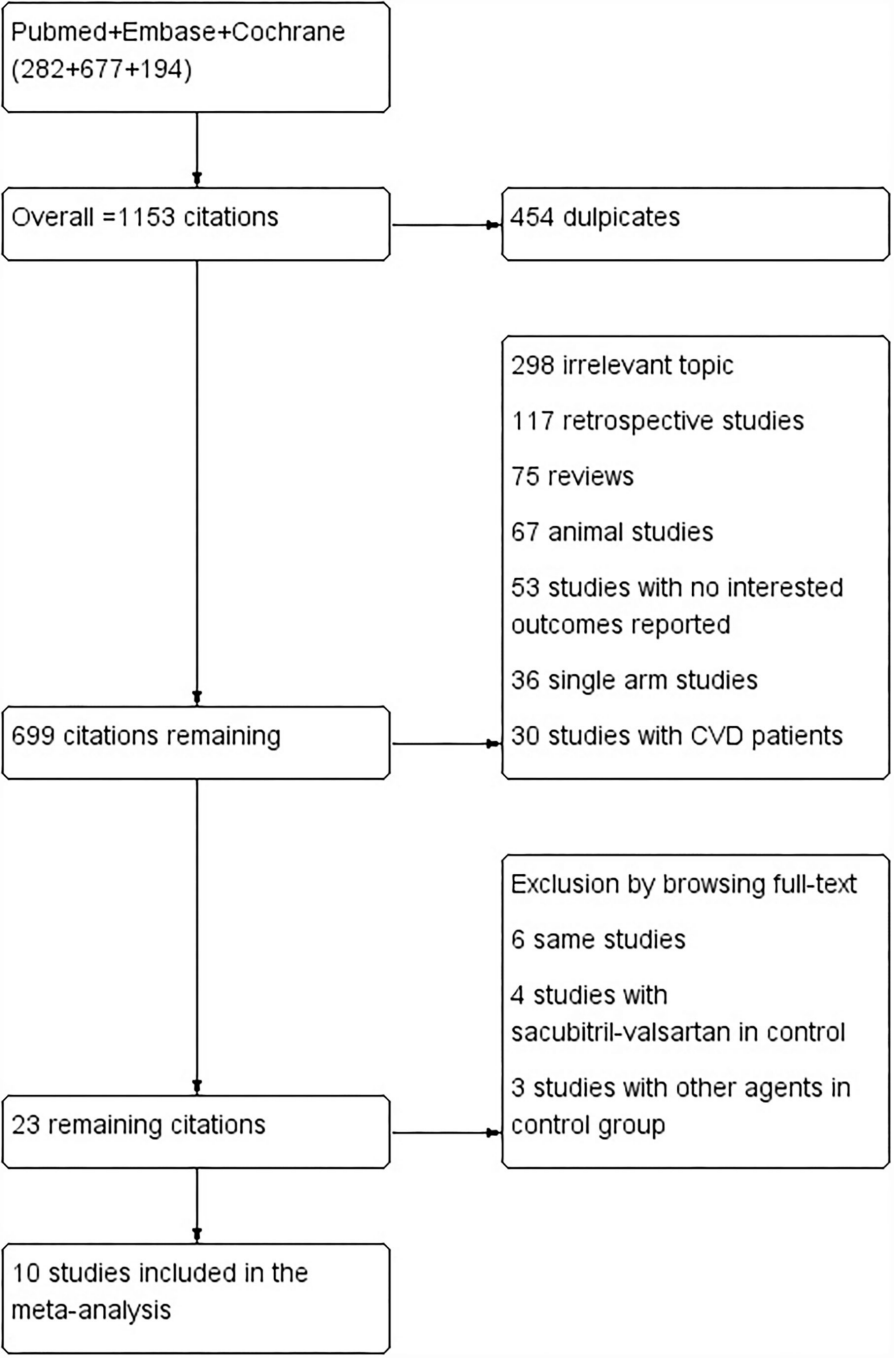
Flowchart diagram of citation screening.

Two authors (Lou and Yu) independently screened eligible studies and extracted the baseline characteristics and outcome data (flowchart in Figure 1). Another two authors independently evaluated the quality of the studies according to the Cochrane Handbook for Systematic Reviews of Interventions (version 5.1.0). Disagreement was resolved by another author (Huang). Incomplete data were obtained by emailing the corresponding author of the study.

#### Outcomes

The primary outcome of the meta-analysis was blood pressure reduction of sacubitril-valsartan 400 mg/day compared with valsartan 320 mg/day. Secondary outcomes were blood pressure reduction of sacubitril-valsartan vs. angiotensin receptor blockers or placebo.

#### Statistical analysis

All the statistical analyses were performed using Review Manager (RevMan, The Cochrane Collaboration, Copenhagen, Denmark) 5.3 software. Mean value and 95% CI were used to compare the efficacy of sacubitril-valsartan vs. other agents. If the heterogeneity across studies was <50%, the fixed effects model was employed, otherwise, the random effects model would be used (15).

### Cost-effectiveness analysis

#### Model structure

The model was developed based on Gu's study of low-cost essential antihypertensive medicines for hypertension control in China (16). In our model, the cost-effectiveness of sacubitril-valsartan vs. other agents was simulated. The starting age of the base case was 60-year-old, consistent with the mean age of patients with hypertension in China (17), and the drugs in both the cohorts were sacubitril-valsartan 400 mg/day and valsartan 320 mg/day. The population in our simulation was those who had hypertension but without established cardiovascular diseases (CVDs) [including coronary heart diseases (CHD), heart failure (HF), and stroke]. The simulation period was a lifetime (until 100-year-old, far higher than the life expectancy in China), and the cycle length was 1 month.

**Figure 2:**
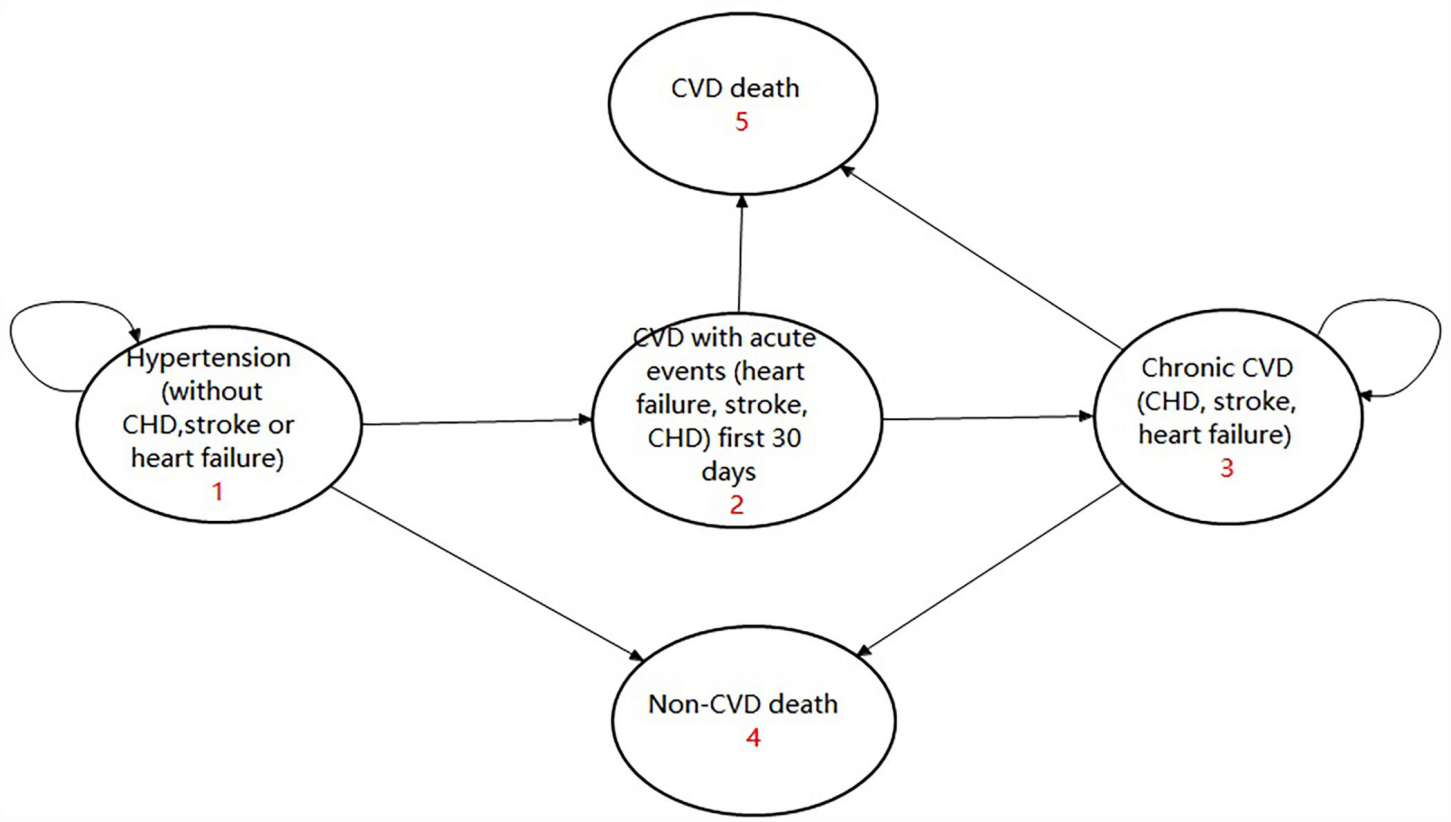
Markov model using state transition diagram.

There were three transition states and two absorbed states in the model, including: (1) Hypertension without CVD, (2) Chronic CVD, (3) CVD with the acute event first 30 days, (4) CVD death, and (5) non-CVD death. A patient with hypertension without CVD might experience an acute CVD events or non-CVD death or he might remain in the state of hypertension. If they experienced an acute CVD event, they would either go to the state of chronic CVD or experience CVD death. Anyone who entered the absorbed state of CVD death or non-CVD death would terminate the cycle. For those who entered the state of chronic CVD, they might experience CVD death or non-CVD death or else would remain in the state of chronic CVD. The model is given in Figure 2 and has been validated by several studies (16, 18).

The present study was conducted from the perspective of the Chinese healthcare system. Only direct costs (drug costs and event costs) were calculated in our analysis with a unit of Chinese Yuan (CNY). The discount rate for future costs and future effectiveness/utility was 0.05 (ranging from 0 to 0.08) according to the China Guidelines for Pharmacoeconomic Evaluations (19). All the statistical analyses were performed using Treeage Pro 2011 software (Williamstown, Massachusetts, USA), and a half-cycle correction was applied to the model to prevent overestimating the costs and effectiveness.

#### Parameter input

##### Transition probability input

The study was performed based on the hypothesis that sacubitril-valsartan was more effective in reducing BP compared with ARB or placebo and the reduction in BP could reduce the CVD incidence. The reduction in BP was derived from the above meta-analysis, and the reduction in risk for CHD, HF, and stroke was accessed from a large-scale meta-analysis, which reported that for those with hypertension but without established CVD, per 5 mm Hg reduction in systolic BP would lead to a hazard ratio (HR) and 95% CI of 0.95 (0.91–0.99), 0.83 (0.77–0.89), and 0.85 (0.80–0.90) for CHD, HF, and stroke, separately (Table 1) (1).

**Table 1:** Model parameters.

**Parameters**	**Value**	**Range_low**	**Range_high**	**Source**
Incidence (event/100 patient*year) ^**a**^				
HF	0.28%	0.25%	0.30%	(20)
Stroke	0.35%	0.33%	0.36%	(21)
CHD	1.02%	/	/	(22)
Mortality (No of death/100 patient*year) ^**b**^				
HF	6.46%	6.10%	6.82%	(23)
Stroke	9.88%	9.21%	10.55%	(21)
CHD	12.18%	/	/	(22)
**Mortality during hospitalization** ^**c**^				
HF	2.80%	2.63%	2.98%	(24)
Stroke	1.56%	1.55%	1.57%	(21)
CHD	2.60%	/	/	(22)
**HR for per 5 mm Hg reduction in systolic BP**				
Stroke	0.85	0.80	0.90	(1)
HF	0.83	0.77	0.89	(1)
CHD	0.95	0.91	0.99	(1)
**Utilities (year)** ^**d**^				
Hypertension	0.96	0.91	1.00	(18)
CHD event	0.6	0.57	0.63	(18)
Stroke event	0.55	0.53	0.58	(18)
HF event	0.63	0.60	0.66	(18)
CHD state	0.7	0.67	0.74	(18)
Stroke state	0.65	0.62	0.68	(18)
HF state	0.73	0.69	0.77	(18)
**Costs of drugs (CNY/month)**				
Sacubitril-valsartan (400 mg/day)	324.6	162.3	649.2	(25)
Sacubitril-valsartan (200 mg/day)	162.3	81.15	324.6	(25)
Sacubitril-valsartan (100 mg/day)	95.4	47.7	190.8	(25)
Valsartan (320 mg/day)	240.0	120.0	480.0	(25)
Olmesartan (40 mg/day)	256.6	128.3	513.2	(25)
Olmesartan (20 mg/day)	128.3	64.2	256.6	(25)
Olmesartan (10 mg/day)	64.2	32.1	128.3	(25)
**Costs of events (CNY/event)** ^**e**^				
Stroke	16,213.6	8,106.8	32,427.2	(26)
HF	9,789.6	4,894.8	19,579.1	(20)
CHD	18,183	9,091.5	36,366.1	(22)
**Annual costs of CVD** ^**f**^				
Stroke	13,265.9	6,632.9	26,531.7	(26)
HF	15,872.4	7,936.2	31,744.8	(20)
CHD	17,644	8,822	35,288	(22)

*HF, heart failure; CHD, coronary heart diseases; HR, hazard ratio; CVD, cardiovascular diseases*.

a Incidence (event/100 patient*year) was converted to transition probabilities with unit of month when inputting these parameters into Markov model. The formula of transformation is 1-month rate = –[ln(1 – incidence)]/12 and 1-month transition probability = 1–exp (−1-month rate).

b Mortality (No. of death/100 patient*year) was converted to transition probabilities with unit of month when inputting these parameters into Markov model. The formula of transformation is 1-month mortality rate = –[ln(1–mortality)]/12 and 1-month transition probability = 1 – exp (−1-month mortality rate).

c *Mortality during hospitalization was converted to transition probabilities when inputting these parameters into Markov model. The formula of transformation is transition probability = 1 – exp (−1 – Mortality)*.

d *Utilities (year) were converted to utilities (month) with Utilities (year)/12 when inputting these parameters into Markov model*.

e,f *Costs were converted to corresponding costs in 2021 in China using healthcare consumer price index (CPI). The CPI from 2015 to 2021 is 1.027, 1.038, 1.06, 1.043, 1.024, 1.018 and 1.004, separately*.

The incidence of CHD was obtained from the Report on Cardiovascular Health and Diseases in China, which reported that the incidence was 1.02%, and the annual mortality rate and in-hospital mortality rate were 12.18 and 2.6%, respectively (22). Incidence (event/year) was converted to transition probability (month) using the formula of 1-month rate = –[ln(1 – incidence)]/12 and 1-month transition probability = 1 – exp (−1-month rate) (27). The transition probability for CHD, death, and in-hospital death was 0.0854, 1.0763, and 2.5665%, respectively. Using the same formula, we could draw that the transition probabilities for HF and stroke were 0.0229 and 0.0288%, and the transition probabilities for death and in-hospital death for HF and stroke were 0.5368 vs. 0.8632% and 2.7683 vs. 1.5447% (Table 1) (20, 21, 23).

##### Costs input

All the drug costs were derived from the price of joint purchasing, launched by the Chinese government (28). The costs of sacubitril-valsartan (400 mg/day per month) were 324.6 CNY = 37.87 (CNY/7 tablet)/7 × 60 (tablet). The costs of sacubitril-valsartan (200 mg/day per month) were 162.3 CNY, half of the costs of 400 mg/day. The costs of sacubitril-valsartan (100 mg/day per month) were 95.4 CNY = 44.52 (CNY/14 tablet)/14 × 30 (tablet). The costs of valsartan (320 mg/day per month) were 240 CNY = 56 (CNY/28 tablet × 80 mg/tablet)/28 × 4 (tablet/day) × 30 (day). The drug price for 20 mg of olmesartan was 4.28 CNY = 29.94 (CNY/7 tablet)/7, and the monthly costs for olmesartan 40, 20, and 10 mg/day were 256.6, 128.3, and 64.2 CNY, respectively (Table 1).

For costs of CHD, HF, and stroke, there were mainly two aspects, including annual costs and events costs. The costs of CHD were derived from the Report on Cardiovascular Health and Diseases in China (22), and the annual costs and event costs were 17,573.7 and 18,110.6 CNY in 2020. The costs for HF and stroke were from published studies conducted in China (20, 26), and the annual costs and event costs for HF were 14,540.4 and 8,968.1 CNY (2017), and 13,213 and 16,149 CNY for stroke (2020). All the costs were converted to the corresponding costs in China in 2021 using the consumer price index (CPI), and the healthcare CPI in China from 2015 to 2021 is 1.027, 1.038, 1.06, 1.043, 1.024, 1.018, and 1.004, respectively (29). For annual costs, they were converted to monthly costs as the cycle length was 1 month (Table 1).

##### Utility input

The utilities for chronic states of hypertension, CHD, HF, and stroke were 0.96, 0.7, 0.73, and 0.65, respectively. For event utility, they were 0.6, 0.63, and 0.55 for CHD, HF, and stroke. All the utilities were acquired from a published study and converted to monthly utilities (Table 1).

##### Outcome

The primary outcome of the present study was the incremental cost-utility ratio (ICUR), which represented the ratio of the incremental cost to the incremental utility. Secondary outcomes were incremental cost-effectiveness ratio (ICER), total costs, incremental costs, life-years (LYs), and quality-adjusted life-years (QALYs). The willingness-to-pay (WTP) was set three times of per capita GDP in China in 2021, which was 242,928 CNY = 80,976 CNY × 3, according to the recommendation of the China Guidelines for Pharmacoeconomic Evaluations (19). Sacubitril-valsartan would be considered cost-effective in treating hypertension if the ICUR was lower than the WTP threshold, otherwise, it would be not cost-effective.

Scenario analyses were also performed based on different control agents (including different doses). The agents in the control group included placebo, olmesartan 40 mg, olmesartan 20 mg, and olmesartan 10 mg. The dose in sacubitril-valsartan also had several doses of 400, 200, and 100 mg. What is more, scenario analysis based on different starting ages was also performed, with 30-year-old, 40-year-old, 50-year-old, and 60-year-old, to simulate the actual hypertension population in China better.

##### Sensitivity analysis

One-way sensitivity analysis and probabilistic sensitivity analysis (PSA) were employed to validate the robustness of our results. In the one-way sensitivity analysis, parameters fluctuated in their 95% CI% or given range. The results of the one-way sensitivity analysis were illustrated with the Tornado diagram. The PSA was performed with 10,000 times Monte Carlo simulation based on probabilistic sensitivity. The cost-effectiveness acceptability curve and scatter plot were drawn to show the results of PSA.

## Results

### Meta-analysis

#### Study selection and baseline characteristics

The keywords search of the PubMed, Embase, and Cochrane databases yielded a total of 1,153 citations. Of which, 454 are duplicates, 298 are irrelevant topics, 117 are retrospective studies, 75 are reviews, 67 are animal studies, 53 studies with interesting outcomes reported, 36 are single-arm studies, and 30 studies with patients with CVD. Finally, 23 remaining citations were searched full text and 13 studies were excluded by browsing full text, and a total of 10 studies of 5,781 patients were included in the meta-analysis (8–10, 13, 30–35). Of the studies included, four studies of 1,038 patients were allocated to the groups of sacubitril-valsartan 400 mg/day or valsartan 320 mg/day, two studies of 554 patients were allocated to the groups of sacubitril-valsartan 400 mg/day or olmesartan 40 mg/day, two studies of 868 patients allocated to the groups of sacubitril-valsartan 400 mg/day or olmesartan 20 mg, four studies of 2,693 patients allocated to the groups of sacubitril-valsartan 200 mg/day or olmesartan 20 mg, three studies of 321 patients allocated to the groups of sacubitril-valsartan 400 mg/day or placebo, one study of 190 patients allocated to the groups of sacubitril-valsartan 200 mg/day or placebo, and one study of 192 patients were allocated to the groups of sacubitril-valsartan 100 mg/day or placebo (Table 2).

**Table 2:** Baseline characteristics of included studies.

**Study name**	**Registration no**.	**Sample size**	**Age**	**Male (%)**	**Intervention (drug, mg/day)**	**Control**	**Race (Mainly)**	**Baseline BP**	**Follow-up period (week)**
Williams et al. (35)	NCT01692301	454	67.7	52.2	Sac-Val, 400	Olme, 40	White	158.6/87.8	52
Wang et al. (9)	NCT01681576	72	57.3	64	Sac-Val, 400	Val, 320	Asian	147.3/90.3	4
Supasyndh et al. (30)	NCT01615198	588	70.7	50	Sac-Val, 200	Olme, 20	Asian	160.3/84.9	10
Schmieder et al. (31)	NCT01870739	114	59.8	67.5	Sac-Val, 400	Olme, 40	White	155.2/92.2	52
Ruilope et al. (10)	/	471	54.8	100	Sac-Val, 400	Val, 320/Placebo	White	158.5/97.0	8
Ruilope et al. (10)	/	376	58.1	0	Sac-Val, 400	Val, 320/Placebo	White	157.1/95.1	8
Rakugi et al. (32)	NCT01599104	1161	58.7	70.5	Sac-Val, 400/200	Olme, 20	Asian	157.9/94.3	8
Kario et al. (33)	NCT01193101	389	51.6	70.7	Sac-Val, 400/200/100	Placebo	Asian	155.0/99.9	8
Izzo et al. (8)	NCT01281306	343	61.5	53.4	Sac-Val, 400	Val, 320/Placebo	White	159.7/90.6	8
Huo et al. (13)	NCT01785472	1438	57.7	52.6	Sac-Val, 400/200	Olme, 20	Asian	158.0/90.4	8
Cheung et al. (34)	NCT01876368	375	57.6	51.2	Sac-Val, 200	Olme, 20	White	157.5/90.8	8

#### Outcomes

For comparison of sacubitril-valsartan 400 mg/day vs. valsartan 320 mg/day, a reduction in BP of −5.97 (−6.38, −5.56) (*p* < 0.01) was observed. For comparison of sacubitril-valsartan (400 mg/day) vs. olmesartan (40 and 20 mg), the reduction in BP was −6.19 (−12.38, −0.01) (*p* = 0.05), −5.25 (−8.64, −1.86) (*p* < 0.01), separately. For comparison of sacubitril-valsartan (200 mg/day) vs. olmesartan (20 and 20 mg), the reduction in BP was −4.53 (−6.54, −2.52) (*p* < 0.01). The reduction in BP was higher when the agent of control is placebo, which was −13.99 (−15.74, −12.24) (*p* < 0.01), −12.57 (−12.94, −12.20) (*p* < 0.01), and −11.86 (−12.22, −11.50) (*p* < 0.01) for sacubitril-valsartan 400, 200, and 100 mg (Table 3).

**Table 3:** Reduction in blood pressure of sacubitril-valsartan compared with other agents.

**Intervention (drug, mg/day)**	**Control (drug, mg/day)**	**Reduction in BP (mmHg)**	**Range**	***P* value**
Sac-Val, 400	Val, 320	−5.97	−6.38, −5.56	<0.01
Sac-Val, 400	Olme, 40	−6.19	−12.38, −0.01	0.05
Sac-Val, 400	Olme, 20	−5.25	−8.64, −1.86	<0.01
Sac-Val, 400	Placebo	−13.99	−15.74, −12.24	<0.01
Sac-Val, 200	Olme, 20	−4.53	−6.54, −2.52	<0.01
Sac-Val, 200	Placebo	−12.57	−12.94, −12.20	<0.01
Sac-Val, 100	Placebo	−11.86	−12.22, −11.50	<0.01

*Sac- Val, sacubitril-valsartan; Val, valsartan; Olme, olmesartan*.

### Cost-effectiveness analysis

#### Base case analysis and scenario analysis

After a simulation of the lifetime horizon, the prescription of sacubitril-valsartan instead of valsartan as an antihypertensive agent led to higher costs and more effectiveness. For a 60-year-old patient with hypertension, if sacubitril-valsartan was prescribed as the antihypertensive agent, he had a life expectancy of 11.91 QALY with costs of 65,066 CNY, and if valsartan was prescribed as the antihypertensive agent, the life expectancy would be 11.82 QALY with costs of 54,769 CNY; thus, an ICUR of 108,622 CNY/QALY was obtained. What is more, if life quality was omitted, the life year (LY) in the sacubitril-valsartan group was 12.7, and it was 12.63 in the valsartan group, resulting in an ICER of 156,820 CNY/LY (Table 4).

**Table 4:** Base case analysis and scenario analysis.

**Scenario (drug, mg/day)**	**Cost**	**Incre-Cost (CNY)**	**QALY**	**IncreQALY**	**LY**	**IncreLY**	**ICER (CNY/LY)**	**ICUR (CNY/QALY)**
Val, 320	54,769		11.82		12.63			
Sac, 400	65,066	10,297	11.91	0.09	12.7	0.07	156,820	108,622
Olme, 40	57,058		11.82		12.63			
Sac, 400	65,220	7,961	11.92	0.1	12.7	0.07	117,189	81,180
Olme, 20	39,393		11.82		12.63			
Sac, 400	65,220	25,827	11.9	0.08	12.69	0.06	444,134	307,528
Placebo	54,769		11.82		12.63			
Sac, 400	17,340	−37,428	12.02	0.2	12.77	0.14	−267,216	−185,780
Olme, 20	39,393		11.82		12.63			
Sac, 200	42,754	3,362	11.89	0.07	12.68	0.05	66,522	46,046
Placebo	21,727		11.82		12.63			
Sac, 200	40,710	18,982	12.01	0.19	12.76	0.13	146,246	101,620
Placebo	21,727		11.82		12.63			
Sac, 100	31,389	9,662	12	0.18	12.76	0.12	78,368	54,435
**Starting age** **=** **50 years old, time horizon** **=** **lifelong**								
Val, 320	63,101		13.58		14.52			
Sac, 400	75,134	12,033	13.73	0.15	14.64	0.12	99,771	79,334
**Starting age** **=** **70 years old, time horizon** **=** **lifelong**								
Val, 320	42,520		9.24		9.87			
Sac, 400	50,441	7,920	9.28	0.04	9.89	0.02	461,281	191,955

*Sac-Val, sacubitril-valsartan; Val, valsartan; Olme, olmesartan*.

Scenario analysis showed that whatever the type of ARB is or whatever the dose of sacubitril-valsartan was, the ICUR was always lower than the WTP of 242,928 CNY/QALY, except for the occasion when sacubitril-valsartan 400 mg/day rather than olmesartan 20 mg/day was prescribed as the antihypertensive agent, which gained an ICUR of 307,528 CNY/QALY, is higher than the WTP of 242,928 CNY/QALY in China. Scenario analysis based on different time horizons yielded similar results that ICUR was lower than the WTP threshold (Table 4).

#### One-way sensitivity analysis

**Figure 3:**
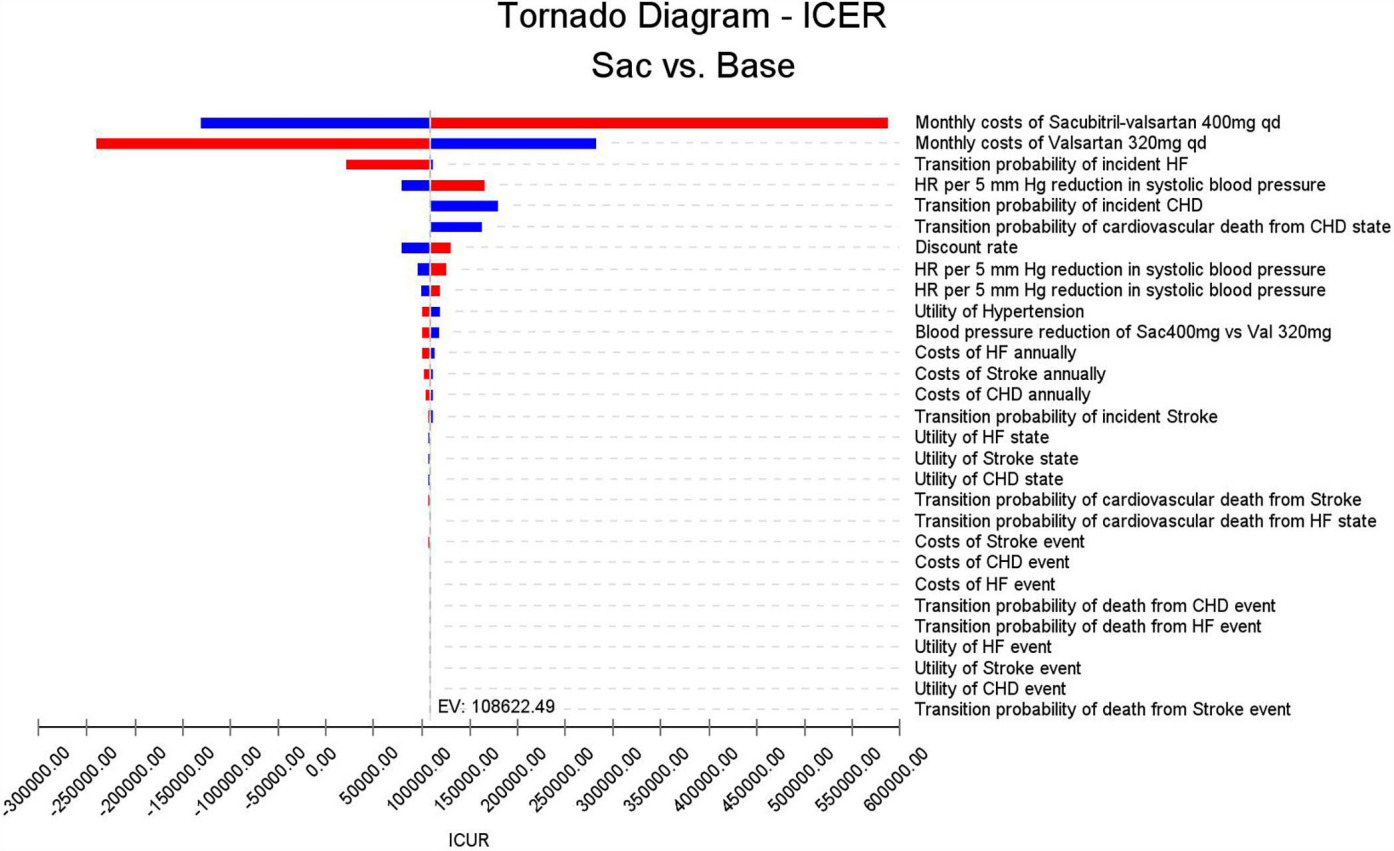
Tornado diagram based on the one-way sensitivity analysis. Costs of sacubitril-valsartan and costs of valsartan impact the largest on the ICUR fluctuation; other input parameters impact little on ICUR.

As can be seen in Figure 3, costs of monthly sacubitril-valsartan impacted the largest on the fluctuation of ICUR, and if the costs of sacubitril-valsartan increased to two times the current price, the ICUR would be higher than the WTP threshold. Similarly, if the costs of valsartan decreased to half of the current price, sacubitril-valsartan would not be cost-effective. Other factors, including other costs, utilities, and transition probabilities, impacted little on the ICUR fluctuation.

#### Probabilistic sensitivity analysis

**Figure 4:**
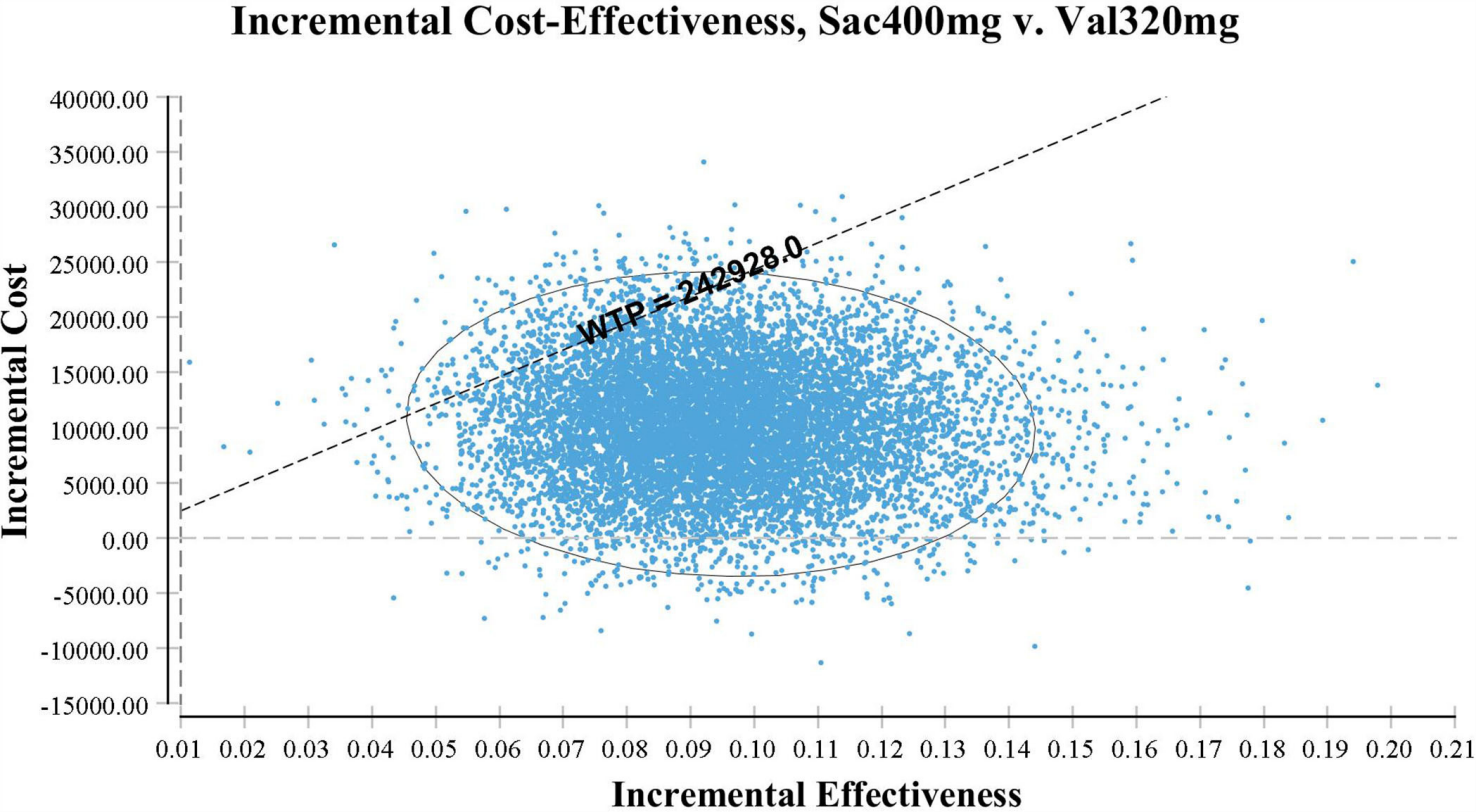
Scatter plot based on probabilistic sensitive analysis. The probability that sacubitril-valsartan is cost-effective or superior to enalapril is over 95%.

**Figure 5:**
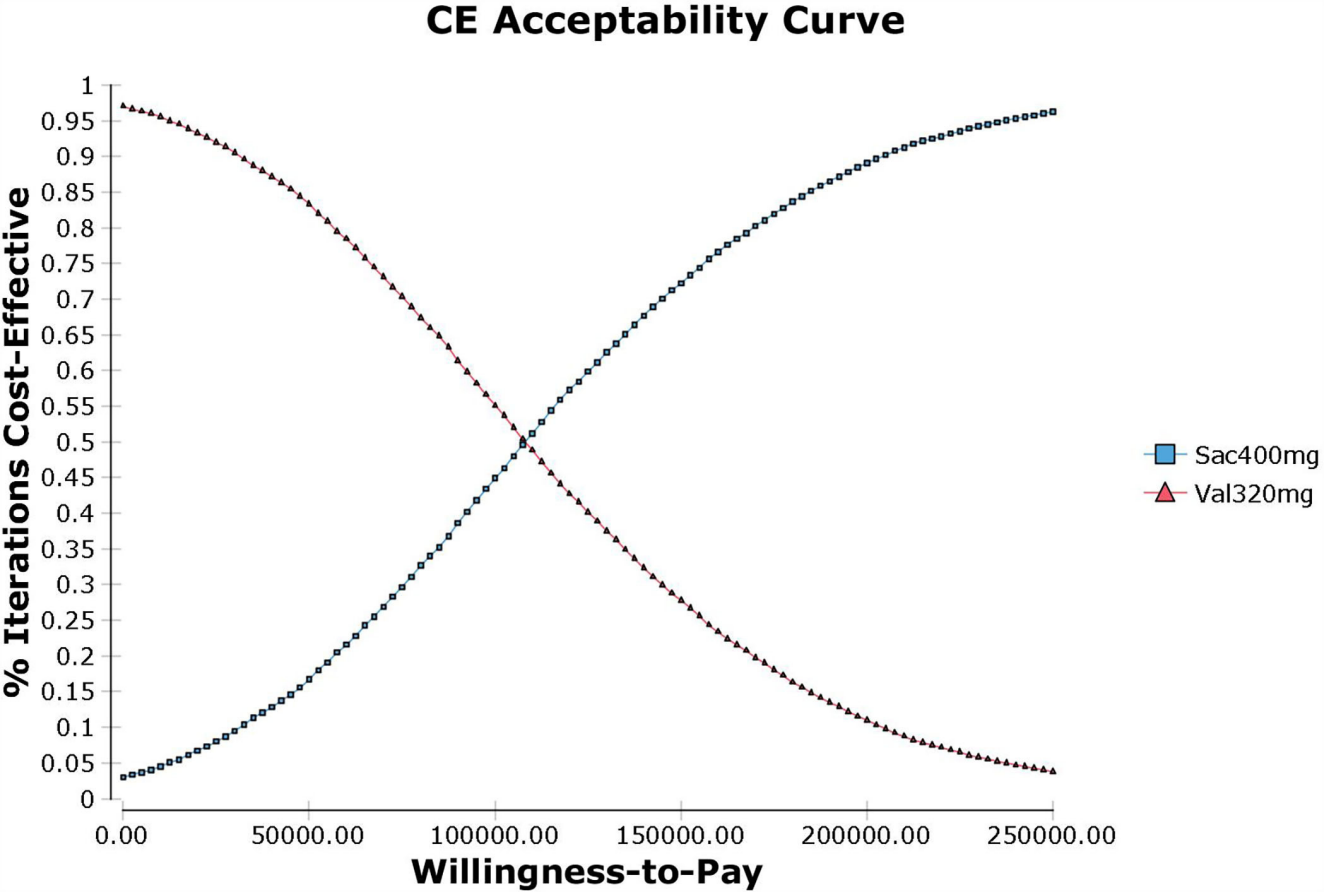
Cost-effectiveness acceptability curve of sacubitril-valsartan vs. valsartan in treating hypertension in China setting. When the WTP is 108,200 CNY/QALY (1.34 times of per capita GDP in China in 2021), sacubitril-valsartan and valsartan share a similar acceptability.

Probabilistic sensitivity analysis using Monte Carlo simulations based on probabilistic sensitivity sampling was conducted to invalidate the robustness of the results. In Figure 4, the scatter plot showed that under 95.56% of circumstances, sacubitril-valsartan was cost-effective in treating hypertension compared with valsartan. The cost-effectiveness acceptability curve also suggested that when the WTP threshold was 108,200 CNY/QALY, sacubitril-valsartan and valsartan shared similar acceptability, and the acceptability of sacubitril-valsartan was higher when the WTP threshold was higher than 108,200 CNY/QALY (Figure 5).

## Discussion

To the best of our knowledge, the present study is the first one to investigate the cost utility of sacubitril-valsartan in treating hypertension and we find that sacubitril-valsartan costs more money and gains more utilities compared with ARB. Sacubitril-valsartan is considered cost-effective in treating hypertension in current China as the ICUR is lower than the WTP threshold.

Sacubitril-valsartan is recommended as the first-line treatment of HF according to the latest ESC heart failure guidelines (6). Nowadays, sacubitril-valsartan has accounted for 63.7% of the renin-angiotensin-aldosterone system inhibitors in patients with HF in the report 2020 Clinical Performance and Quality Measures for Heart Failure in China (24). However, sacubitril-valsartan is not widely used in patients with hypertension; it may attribute to several aspects. First, sacubitril-valsartan is not recommended for hypertension in previous guidelines. The Chinese expert recommendation of sacubitril-valsartan for hypertension is the first to recommend sacubitril-valsartan as an antihypertensive agent (12, 36). Second, the price of sacubitril-valsartan is higher than the other antihypertensive agents, and whether the costs are worth the efficacy is still unclear. The effectiveness of sacubitril-valsartan on antihypertension is inconsistent, and the reduction in BP ranges from 2.33 to 15.38 compared with other antihypertensive agents (including placebo) (13, 33). To investigate the real effect of sacubitril-valsartan on antihypertension, a meta-analysis based on RCTs of hypertension without established CVD is necessary. In our meta-analysis, we found that compared with ARB, the antihypertensive agent could reduce about 5 mm Hg in BP. In view that sacubitril-valsartan is the alternative to ARB, the cost-utility analysis is mainly based on the comparison of sacubitril-valsartan vs. ARB.

In a study investigating low-cost essential antihypertensive medicines for hypertension control in China, Gu et al. found that low-cost essential antihypertensive medicines were cost-effective in reducing complications of hypertension (16). However, another study conducted in China found that drug treatment was not cost-effective compared with non-drug for patients with stage I hypertensive aged ≥65 years without cardiovascular disease in China (37). These results suggest that high-cost antihypertensive medicine may not be cost-effective in the current China setting, as the purpose of treating hypertension is to prevent complications, and the incidence of complications is not so high. In our study, this finding is proven by our one-way sensitivity analysis. In the Tornado diagram, we can find that the costs of sacubitril-valsartan and costs of valsartan impacted the largest on the ICUR fluctuation, suggesting that costs of antihypertensive agents were the main factors of cost-effectiveness.

Besides the costs of sacubitril-valsartan, WTP threshold is another factor of cost-effectiveness. Due to the differences in economic development between various countries or regions, the WTP threshold varies a lot in different countries, especially between developed countries and developing counties. The WTP threshold in China is 242,928 CNY/QALY, equal to 37,655 US$/QALY, and the value is 5,089 US$/QALY in Thailand (27), 45,500 in Japan (38), 79,241 in Germany (39), and 100,000 in the USA (40). Given the differences in WTP among countries, the price of sacubitril-valsartan varies among countries. It could be found that the WTP threshold is higher in Australia than in Thailand, but studies found that sacubitril-valsartan is cost-effective for acute decompensated heart failure in Thailand but not cost-effective in Australia (27, 41). These findings suggest that our conclusion could only be extrapolated to countries or regions with similar conditions to China.

Even though sacubitril-valsartan has been proven cost-effective in patients with HF in China (42), adding sacubitril-valsartan to the standard treatment of hypertension still needs investigating. In our study, we found that sacubitril-valsartan was cost-effective in treating hypertension in the current China setting, despite starting age or time horizon. This is partly due to the policy of joint purchasing launched by the Chinese government to provide better healthcare. Sacubitril-valsartan was included in the lists of joint purchasing in 2021, and the price of sacubitril-valsartan has decreased to about one-third of the initial price. In our one-way sensitivity analysis, we found that if the costs of sacubitril-valsartan increase to two times the current price, sacubitril-valsartan would be not cost-effective, and these results indicated that the current price of sacubitril-valsartan is acceptable in China.

It is reported that the prevalence of hypertension in China is 27.9%, and about 2.54 million people die of hypertension every year (22). Hypertension poses a huge burden on China and low-cost antihypertensive agents are necessary. Currently, common antihypertensive including β-blockers, RAASi, diuretics, and calcium channel blockers are at a low cost. Though the costs of sacubitril-valsartan are higher than ARB, our study demonstrated that sacubitril-valsartan is still cost-effective in Chinese patients with hypertension.

Sacubitril-valsartan was approved to treat heart failure by the China National Medical Products Administration (NMPA) in 2017 (42); the proportion of sacubitril-valsartan in RAASi was 2.3% at that time, but it has increased to 63.7% in 2020 as sacubitril-valsartan was included in the joint purchasing list (24). Sacubitril-valsartan was recommended as antihypertensive drug by the Chinese expert recommendations in 2021, and was approved to treat hypertension by the NMPA a few months later (12). In the same year, the indications for the treatment of hypertension with sacubitril-valsartan were included in the National Medical Insurance Catalog of 2021 (25). Even though the proportion of sacubitril-valsartan in RAASi is unclear currently, the approved indications for the treatment of hypertension will make it more widely available.

There are several limitations to our study. First, the present study is based on the mathematical model; a real-world study may provide better evidence, even though the deterministic analysis and uncertain analysis both validate the robustness of our results. Second, the study was conducted using Chinese domestic data; it may not be extrapolated to patients with hypertension in other regions. Third, the study is performed from the perspective of the healthcare providers; indirect costs are not included. This limited us to analyze it from society's perspective, which is the most comprehensive perspective.

## Conclusion

Compared with valsartan, sacubitril-valsartan is more effective in reducing blood pressure and may result in more life years and quality-adjusted life-years, although with higher costs. Sacubitril-valsartan is cost-effective for hypertension in current China setting under the willingness-to-pay threshold of 3 times of per capita GDP.

## Data availability statement

The original contributions presented in the study are included in the article/Supplementary material, further inquiries can be directed to the corresponding author/s.

## Author contributions

JH came up with the idea and designed the protocol. YL synthesized the data and drafted the manuscript. YY and JL participated in the data collection and data analysis. All authors have approved the final version of the manuscript.

## Conflict of interest

The authors declare that the research was conducted in the absence of any commercial or financial relationships that could be construed as a potential conflict of interest.

## Publisher's note

All claims expressed in this article are solely those of the authors and do not necessarily represent those of their affiliated organizations, or those of the publisher, the editors and the reviewers. Any product that may be evaluated in this article, or claim that may be made by its manufacturer, is not guaranteed or endorsed by the publisher.
