# Supplementary material for: Sacubitril-valsartan for the treatment of hypertension in China: A cost-utility analysis based on meta-analysis of randomized controlled trials
Source: Front Public Health. 2022 Aug 17;10:959139. doi: 10.3389/fpubh.2022.959139 (PMC9432800; doi:10.3389/fpubh.2022.959139)
Supplement: Supplementary file 1 [file Table_1.DOC]

**Search strategy for PubMed**

Search number Query Sort By Filters Search Details Results Time

12 ((((((("sacubitril and valsartan sodium hydrate drug combination" [Supplementary Concept]) OR ("Neprilysin"[Mesh])) OR (Sacubitril[Title/Abstract])) OR (LCZ696[Title/Abstract])) OR (Angiotensin Receptor Neprilysin Inhibition[Title/Abstract])) OR (Angiotensin Neprilysin Inhibition[Title/Abstract])) AND ((blood pressure[Title/Abstract]) OR (hypertension[Title/Abstract]))) AND ((clinical[tiab] AND trial[tiab]) OR "clinical trials as topic"[mesh] OR "clinical trial"[pt] OR random*[tiab] OR "random allocation"[mesh] OR "therapeutic use"[sh]) Most Recent ("sacubitril and valsartan sodium hydrate drug combination"[Supplementary Concept] OR "Neprilysin"[MeSH Terms] OR "Sacubitril"[Title/Abstract] OR "LCZ696"[Title/Abstract] OR "angiotensin receptor neprilysin inhibition"[Title/Abstract] OR "angiotensin neprilysin inhibition"[Title/Abstract]) AND ("blood pressure"[Title/Abstract] OR "hypertension"[Title/Abstract]) AND (("clinical"[Title/Abstract] AND "trial"[Title/Abstract]) OR "clinical trials as topic"[MeSH Terms] OR "clinical trial"[Publication Type] OR "random*"[Title/Abstract] OR "random allocation"[MeSH Terms] OR "therapeutic use"[MeSH Subheading]) 407 23:09:55

11 (clinical[tiab] AND trial[tiab]) OR "clinical trials as topic"[mesh] OR "clinical trial"[pt] OR random*[tiab] OR "random allocation"[mesh] OR "therapeutic use"[sh] Most Recent ("clinical"[Title/Abstract] AND "trial"[Title/Abstract]) OR "clinical trials as topic"[MeSH Terms] OR "clinical trial"[Publication Type] OR "random*"[Title/Abstract] OR "random allocation"[MeSH Terms] OR "therapeutic use"[MeSH Subheading] 6,042,272 23:08:46

10 (blood pressure[Title/Abstract]) OR (hypertension[Title/Abstract]) Most Recent "blood pressure"[Title/Abstract] OR "hypertension"[Title/Abstract] 651,855 23:00:02

9 hypertension[Title/Abstract] Most Recent "hypertension"[Title/Abstract] 433,030 22:59:49

8 blood pressure[Title/Abstract] Most Recent "blood pressure"[Title/Abstract] 329,166 22:59:33

7 ((((("sacubitril and valsartan sodium hydrate drug combination" [Supplementary Concept]) OR ("Neprilysin"[Mesh])) OR (Sacubitril[Title/Abstract])) OR (LCZ696[Title/Abstract])) OR (Angiotensin Receptor Neprilysin Inhibition[Title/Abstract])) OR (Angiotensin Neprilysin Inhibition[Title/Abstract]) Most Recent "sacubitril and valsartan sodium hydrate drug combination"[Supplementary Concept] OR "Neprilysin"[MeSH Terms] OR "Sacubitril"[Title/Abstract] OR "LCZ696"[Title/Abstract] OR "angiotensin receptor neprilysin inhibition"[Title/Abstract] OR "angiotensin neprilysin inhibition"[Title/Abstract] 6,442 22:58:59

6 Angiotensin Neprilysin Inhibition[Title/Abstract] Most Recent "angiotensin neprilysin inhibition"[Title/Abstract] 33 22:58:37

5 Angiotensin Receptor Neprilysin Inhibition[Title/Abstract] Most Recent "angiotensin receptor neprilysin inhibition"[Title/Abstract] 57 22:58:08

4 LCZ696[Title/Abstract] Most Recent "LCZ696"[Title/Abstract] 299 22:57:49

3 Sacubitril[Title/Abstract] Most Recent "Sacubitril"[Title/Abstract] 1,315 22:57:31

2 "Neprilysin"[Mesh] Most Recent "Neprilysin"[MeSH Terms] 5,271 22:41:15

1 "sacubitril and valsartan sodium hydrate drug combination" [Supplementary Concept] Most Recent "sacubitril and valsartan sodium hydrate drug combination"[Supplementary Concept] 919 22:37:40

**Search strategy for Embase**

No. Query Results Results Date

#12. #7 AND #10 AND #11 704 10 May 2022

#11. 'clinical':ti,ab AND 'trial':ti,ab OR 'clinical 6,504,582 10 May 2022

trial'/exp OR random* OR 'drug therapy':lnk

#10. #8 OR #9 985,655 10 May 2022

#9. 'hypertension':ti,ab,kw 685,479 10 May 2022

#8. 'blood pressure':ti,ab,kw 469,836 10 May 2022

#7. #1 OR #2 OR #3 OR #4 OR #5 OR #6 6,090 10 May 2022

#6. angiotensin AND neprilysin AND 564 10 May 2022

inhibition:ti,ab,kw

#5. angiotensin AND receptor AND neprilysin AND 465 10 May 2022

inhibition:ti,ab,kw

#4. lcz696:ti,ab,kw 529 10 May 2022

#3. neprilysin:ti,ab,kw 3,344 10 May 2022

#2. sacubitril:ti,ab,kw 2,483 10 May 2022

#1. 'sacubitril plus valsartan'/exp 3,639 10 May 2022

**Search strategy for Cochrane**

ID Search Hits

#1 MeSH descriptor: [Neprilysin] explode all trees 173

#2 (sacubitril and valsartan sodium hydrate drug combination):ti,ab,kw (Word variations have been searched) 5

#3 (Neprilysin):ti,ab,kw (Word variations have been searched) 410

#4 (Angiotensin Receptor Neprilysin Inhibition):ti,ab,kw (Word variations have been searched) 77

#5 (LCZ696):ti,ab,kw (Word variations have been searched) 236

#6 (Angiotensin Neprilysin Inhibition):ti,ab,kw (Word variations have been searched) 119

#7 #1 or #2 or #3 #4 or #5 or #6 431

#8 (blood pressure):ti,ab,kw (Word variations have been searched) 114057

#9 (hypertension):ti,ab,kw (Word variations have been searched) 70331

#10 #8 or #9 149841

#11 #7 and #10 196

CHEERS 2022 Checklist

|  | **Item** | **Guidance for Reporting** | **Reported in section** |
| --- | --- | --- | --- |
| **TITLE** | | | P 1 |
| Title | 1 | Identify the study as an economic evaluation and specify the interventions being compared. | Line 1 |
| **ABSTRACT** | | | P 2 |
| Abstract | 2 | Provide a structured summary that highlights context, key methods, results and alternative analyses. | Line 1 |
| **INTRODUCTION** | | | P 3 |
| Background and objectives | 3 | Give the context for the study, the study question and its practical relevance for decision making in policy or practice. | Line 1-23 |
| **METHODS** | | | P4-6 |
| Health economic  analysis plan | 4 | Indicate whether a health economic analysis plan was developed and  where available. | P4, Line 14 |
| Study population | 5 | Describe characteristics of the study population (such as age range, demographics, socioeconomic, or clinical characteristics). | P3, Line 33 |
| Setting and location | 6 | Provide relevant contextual information that may influence findings. | P4, Line 18 |
| Comparators | 7 | Describe the interventions or strategies being compared and why chosen. | P4, Line 17 |
| Perspective | 8 | State the perspective(s) adopted by the study and why chosen. | P4, Line 33 |
| Time horizon | 9 | State the time horizon for the study and why appropriate. | P4, Line 22 |
| Discount rate | 10 | Report the discount rate(s) and reason chosen. | P4, Line 35 |
| Selection of outcomes | 11 | Describe what outcomes were used as the measure(s) of benefit(s) and harm(s). | P5 Line 35 |
| Measurement of outcomes | 12 | Describe how outcomes used to capture benefit(s) and harm(s) were measured. | P5 Line 36 |
| Valuation of outcomes | 13 | Describe the population and methods used to measure and value outcomes. | P5 Line 36 |
| Measurement and valuation of resources  and costs | 14 | Describe how costs were valued. | P5 Line 28 |
| Currency, price date, and conversion | 15 | Report the dates of the estimated resource quantities and unit costs, plus the currency and year of conversion. | P5 Line 25 |
| Rationale and  description of model | 16 | If modelling is used, describe in detail and why used. Report if the model  is publicly available and where it can be accessed. | P4, Line 15 |
| Analytics and assumptions | 17 | Describe any methods for analysing or statistically transforming data, any extrapolation methods, and approaches for validating any model used. | P4, Line 37 |
| Characterizing heterogeneity | 18 | Describe any methods used for estimating how the results of the study vary for sub-groups. | P6, Line 3 |
| Characterizing  distributional effects | 19 | Describe how impacts are distributed across different individuals  or adjustments made to reflect priority populations. | P6, Line 3 |
| Characterizing uncertainty | 20 | Describe methods to characterize any sources of uncertainty in the analysis. | P6, Line 7 |
| Approach to engagement with patients and others affected by the study | 21 | Describe any approaches to engage patients or service recipients, the general public, communities, or stakeholders (e.g., clinicians or payers) in the design of the study. | P3, Line 44 |
| **RESULTS** | | | P6-7 |
| Study parameters | 22 | Report all analytic inputs (e.g., values, ranges, references) including uncertainty or distributional assumptions. | P6, Line12-38  P14-17 |
| Summary of main results | 23 | Report the mean values for the main categories of costs and outcomes of interest and summarise them in the most appropriate overall measure. | P6, Line 40-P7, Line 23 |
| Effect of uncertainty | 24 | Describe how uncertainty about analytic judgments, inputs, or projections  affect findings. Report the effect of choice of discount rate and time horizon, if applicable. | P7, Line 15-23 |
| Effect of engagement with patients and others affected by the study | 25 | Report on any difference patient/service recipient, general public, community, or stakeholder involvement made to the approach or findings of the study | P7, Line 8 |
| **DISCUSSION** | | | P7 |
| Study findings, limitations, generalizability, and current knowledge | 26 | Report key findings, limitations, ethical or equity considerations not captured, and how these could impact patients, policy, or practice. | P7, Line 25-P9, Line 8 |
| **OTHER RELEVANT INFORMATION** | | | |
| Source of funding | 27 | Describe how the study was funded and any role of the funder in the identification, design, conduct, and reporting of the analysis | P10, Line 9 |
| Conflicts of interest | 28 | Report authors conflicts of interest according to journal or  International Committee of Medical Journal Editors requirements. | P10, Line 21 |

Husereau D, Drummond M, Augustovski F, de Bekker-Grob E, Briggs AH, Carswell C, Caulley L, Chaiyakunapruk N, Greenberg D, Loder E, Mauskopf J, Mullins CD, Petrou S, Pwu RF, Staniszewska S; CHEERS 2022 ISPOR Good Research Practices Task Force. Consolidated Health Economic Evaluation Reporting Standards 2022 (CHEERS 2022) Statement: Updated Reporting Guidance for Health Economic Evaluations. BMJ. 2022;376:e067975.

The checklist is Open Access distributed in accordance with the terms of the Creative Commons Attribution (CC BY 4.0) license, which permits others to distribute, remix, adapt and build upon this work, for commercial use, provided the original work is properly cited. See: [http://creativecommons.org/licenses/by/4.0/.](http://creativecommons.org/licenses/by/4.0/)


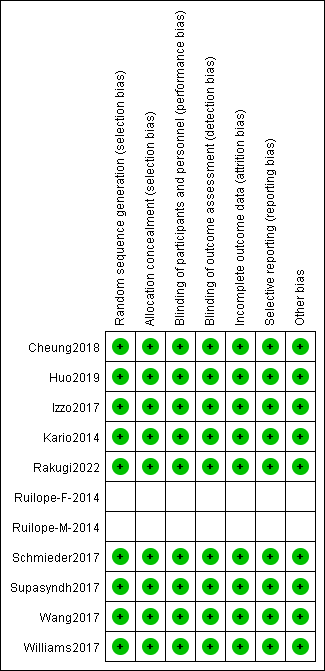


Caption: Risk of bias summary: review authors' judgements about each risk of bias item for each included study.
